# *Cenostigma bracteosum* Hydroethanolic Extract: Chemical Profile, Antibacterial Activity, Cytotoxicity, and Gel Formulation Development

**DOI:** 10.3390/pharmaceutics17060780

**Published:** 2025-06-14

**Authors:** Addison R. Almeida, Francisco A. S. D. Pinheiro, Marília G. M. Fideles, Roberto B. L. Cunha, Vitor P. P. Confessor, Kátia N. Matsui, Weslley S. Paiva, Hugo A. O. Rocha, Gislene Ganade, Laila S. Espindola, Waldenice A. Morais, Leandro S. Ferreira

**Affiliations:** 1 Programa de Pós-Graduação em Química, Instituto de Química, Universidade Federal do Rio Grande do Norte (UFRN), Natal CEP 59072-970, Brazil; addison.ribeiro@ufrn.br; 2 Programa de Pós-Graduação em Ciências Farmacêuticas, Departamento de Farmácia, Universidade Federal do Rio Grande do Norte (UFRN), Natal CEP 59012-570, Brazil; ayrtonsennap@gmail.com (F.A.S.D.P.); vitorconfessor@gmail.com (V.P.P.C.); waldenice.lima@ufrn.br (W.A.M.); 3 Departamento de Farmácia, Universidade Federal do Rio Grande do Norte (UFRN), Natal CEP 59012-570, Brazil; gabimf_@outlook.com (M.G.M.F.); brunolucena.rn@gmail.com (R.B.L.C.); 4 Programa de Pós-Graduação em Engenharia Química, Departamento de Engenharia Química, Universidade Federal do Rio Grande do Norte (UFRN), Natal CEP 59012-570, Brazil; katiamatsui@gmail.com; 5 Laboratório de Biotecnologia de Polímeros Naturais-BIOPOL, Departamento de Bioquímica, Universidade Federal do Rio Grande do Norte (UFRN), Natal CEP 59072-970, Brazil; wdspaiva@gmail.com (W.S.P.); hugo.rocha@ufrn.br (H.A.O.R.); 6 Programa de Pós-Graduação em Ecologia, Departamento de Ecologia, Centro de Biociências, Universidade Federal do Rio Grande do Norte (UFRN), Natal CEP 59072-970, Brazil; gganade@gmail.com; 7 Laboratório de Farmacognosia, Campus Universitário Darcy Ribeiro, Universidade de Brasília, Brasília CEP 70910-900, Brazil; darvenne@gmail.com

**Keywords:** *Cenostigma bracteosum*, Caatinga biome, gel formulation, carbopol, antibacterial, UHPLC-MS/MS

## Abstract

**Background:***Cenostigma bracteosum* (Tul.) Gagnon & G.P. Lewis (Fabaceae), popularly known as “catingueira”, is a plant widely distributed in the Caatinga biome, which comprises 11% of the Brazilian territory. While this species is of interest given local knowledge, formal reports are lacking in the literature, warranting targeted investigation. This study aimed to prepare and characterize a hydroethanolic extract of *C. bracteosum* leaves, prepare carbopol gels containing the extract, and evaluate their cytotoxicity and antibacterial activity against *Staphylococcus aureus* and *Escherichia coli*. **Methods**: The initial extract was prepared in an ultrasonic bath using ethanol/water (70:30, *v*/*v*). The extract (1 mg/mL) was analyzed by liquid chromatography coupled with mass spectrometry (UHPLC-MS/MS). Carbopol-based gels containing 1% and 3% of *C. bracteosum* extract were prepared and characterized in terms of pH, conductivity, spreadability, and rheology. The cytotoxicity was determined by the MTT method using MC3T3-E1 pre-osteoblast cells and L929-CCL1 fibroblast cells. The antibacterial activity of the extract and gels was evaluated using the agar diffusion method against *S. aureus* and *E. coli*. **Results**: The *C. bracteosum* leaves extract demonstrated antibacterial activity against *S. aureus* and *E. coli*, were not cytotoxic for the assessed cells at concentrations up to 100 μg/mL, and its analysis by UHPLC-MS/MS allowed the annotation of 18 metabolites, mainly of the phenolic acid and flavonoids glycoside classes, together with a biflavonoid. The prepared gels remained stable over the 30-day post-production analysis period. **Conclusions**: These findings provide a better understanding of the chemical diversity of the secondary metabolites of a common Caatinga biome species—*C. bracteosum*—specifically present in leaves hydroethanolic extract and gel formulation adapted for skin application with activity against *S. aureus.*

## 1. Introduction

*Cenostigma bracteosum* (Tul.) Gagnon & G.P. Lewis (Fabaceae), popularly known as “catingueira” or “catingueira-brilhante”, is a plant widely distributed in the Caatinga biome, which comprises 11% of the Brazilian territory [1]. While this species is of interest due to local knowledge of its use in treating various health conditions, such as kidney infections, hypertension, diarrhea, hepatitis, and anemia, using the leaves and stem bark [2], formal reports are lacking in the literature, warranting targeted investigation.

There are reports in the literature regarding the antibacterial activity of a *C. bracteosum* methanolic extract against *Staphylococcus saprophyticus* and *Pseudomonas aeruginosa* strains [3]. Plant species of the *Cenostigma* genus are known to produce phenolic compounds and biflavonoids, such as amentoflavone, found in the methanolic extract of *C. pyramidalis* leaves [4]. Therefore, further studies are needed to investigate other biological activities, the chemical composition, and the development of formulations.

Topical administration of extracts containing molecules with antibacterial activity may offer several advantages, including the controlled release of these bioactive molecules, control of first-pass metabolism, and improved adherence to a potential treatment, thereby achieving local or systemic effects [5]. Gels are the most acceptable of all topical formulations. They have better drug-release properties but are more commonly used for hydrophilic molecules or compositions. For the administration of hydrophobic drugs in gel form, a combination of gel and emulsion, known as an emulgel, is typically used [6].

Carbopol polymers were the first commercial carbomers created and have since been widely used as excipients in the pharmaceutical and cosmetic fields due to their broad range of applications. Such polymers are derived from polyacrylic acid, which forms a gel when neutralized in an aqueous solution with a basic pH. Due to the hydrophilic nature of the polymer’s functional groups, the cross-linked structures of carbopol bases render them potential candidates for incorporation into aqueous extracts and polar organic solvents such as ethanol and methanol [7]. They have a high molecular weight and exhibit differences in cross-linking density and viscosity profile depending on the polymer chain [8]. There are reports of work with extracts, oil resins, and nanoparticles, among others, incorporated into carbopol gels with proven results regarding antibacterial activity against *S. aureus*, dermatitis treatment, and permeation studies [8,9,10].

This study aimed to prepare and characterize the hydroethanolic extract of *C. bracteosum*, prepare carbopol gels containing this extract, and evaluate their antibacterial activity against *S. aureus* and *E. coli* and cytotoxicity in L929 fibroblast and MC3T3 pre-osteoblast cells. Additionally, gel characterization and preliminary stability tests were conducted to evaluate pH, conductivity, spreadability, and rheology parameters.

## 2. Materials and Methods

### 2.1. Plant Material

*C. bracteosum* leaves were collected in July 2022 in the Assu National Forest (FLONA), in the semi-arid region of Rio Grande do Norte–Brazil (latitude: 5°34′55″ S, longitude: 36°56′40″ W), a voucher specimen (Ferreira, L.S. 11) was identified and deposited at the Herbarium of the Federal University of Rio Grande do Norte (UFRN), under the number UFRN28823. The species collected was previously registered in the National System for the Management of Genetic Heritage and Associated Traditional Knowledge/SisGen (A8772EF, AC3F99B) under a collection permit in the System of Authorization and Information on Biodiversity/SISBIO (83600). Samples were collected, transported under refrigeration, and stored in a freezer at −20 °C until extract preparation.

### 2.2. Hydroethanolic Extract Preparation

The collected leaf samples were selected, dried in a circulating air oven at 50 °C, ground, and passed through 500 µm sieves. The dried material was then weighed, and solid–liquid extraction was performed in an ultrasonic bath (Quimis Q3350, Diadema, Brazil) for 30 min, using ethanol/water (70:30, *v*/*v*) as the solvent, at a ratio of 10:1 (mg/mL) dry plant drug/solvent. After extraction, samples were filtered, evaporated under reduced pressure using a rotary evaporator at ≤40 °C, frozen at −80 °C for 24 h, and then lyophilized for 48 h.

### 2.3. UHPLC-MS/MS Analysis of the C. bracteosum Hydroethanolic Extract

The *C. bracteosum* hydroethanolic extract (1 mg/mL) was analyzed by liquid chromatography coupled with mass spectrometry (UHPLC-MS/MS; Bruker Daltonics, Bremen, Germany). The system consists of an Elute UHPLC with a diode array detector (DAD) coupled to a Compact model sequential quadrupole-time of flight (Q-TOF) mass spectrometer. A Kinetex C18 column (150 mm length × 4.6 mm i.d. × 2.6 µm particle size), Phenomenex (Torrance, CA, USA), was used with acetonitrile (B) and purified water (A) as the mobile phase, containing 0.1% formic acid (Sigma-Aldrich, St. Louis, MO, USA) in both phases. The injection volume was 5 µL, and gradient mode was 5% of phase B for 1 min, 5–100% in 22 min, maintained at 100% for 2 min and returned to initial condition in 1 min, and maintained at a flow rate of 0.8 mL/min for 4 min. Chromatographic analysis time was 30 min. Sodium formate (10 mM), Merck (Darmstadt, Germany) was used as the calibration solution.

The sample was ionized by electrospray ionization (ESI) using the following parameters—capillary voltage: 4.5 kV, source temperature: 220 °C, drying gas flow rate: 9 L/min, operated in positive mode, mass detection range: *m*/*z* 50 to 1300 Da. Data acquisition was performed using Data Analysis Software (version 6.1), and raw files were converted to the .mzXML format. The files were processed in MZmine 3.9.0 using the following parameters: mass detection with a minimum intensity of 2 × 10^3^ for MS^1^ and 1 × 10^2^ for MS^2^; chromatogram construction used the ADAP chromatogram builder algorithm, considering a retention time of 1 to 21 min, a minimum number of 5 scans, a minimum intensity of 2 × 10^2^*,* and a tolerance of 10 ppm or *m*/*z* 0.005. The processed data were imported into the GNPS2 (https://gnps2.org/homepage) and Sirius 6 platforms to obtain the molecular annotation of the compounds present in the *C. bracteosum* extract. In addition, a literature search was conducted for compounds previously reported from the *Cenostigma* genus.

### 2.4. Cell Culture and Cytotoxicity of Cenostigma bracteosum Extract

The cytotoxicity of *Cenostigma bracteosum* hydroethanolic extract was determined by the MTT method [11] using MC3T3-E1 pre-osteoblast cells and L929-CCL1 fibroblast cells. Cells were grown in culture flasks in α-MEM (MC3T3) or DMEM (L929) medium containing 10% (*v*/*v*) fetal bovine serum (Cultilab, Campinas, Brazil) with 100 μg/mL streptomycin (Cultilab, Campinas, Brazil) and 100 IU/mL penicillin. The cells were cultured in sterile 96-well plates at a density of 5 × 10^3^ cells/well and incubated for 24 h at 37 °C in 5% CO_2_. After that, serum-free culture medium was added to synchronize all cells in the G0 cell cycle phase (active but not dividing) and remained in this state for 24 h. The medium was then removed and replaced with a medium containing fetal bovine serum (10%) and *C. bracteosum* extract (10, 25, 50, and 100 μg/mL). *C. bracteosum* extract was in a stock solution in DMSO (Fagron, Jundiaí, Brazil), and the DMSO concentration did not exceed 1%. The control groups consisted of cells treated with 1% DMSO (DMSO) and cells without DMSO and samples (NT). After 24 h of incubation, the cells were washed with sodium phosphate buffer (PBS). Serum-free culture medium containing 12 mM MTT (3-(4,5-dimethylthiazol-2-yl)-2,5-diphenyltetrazolium bromide) was added, and the cells were incubated for 4 h at 37 °C and 5% CO_2_. To solubilize the reduced MTT product, 100 μL ethanol was added to each well. At 15 min after ethanol addition, the samples were read at a wavelength of 570 nm using a microplate reader (Thermo Labsystems, Franklin, MA, USA).

### 2.5. Preparation of Carbopol-Based Gels Containing the C. bracteosum Extract

Carbopol-based gels were prepared using 1% carbopol 940 (Fagron, Jundiaí, Brazil), 0.1% EDTA (Dinâmica, Indaiatuba, Brazil), and 0.2% methylparaben in distilled water. The methylparaben (Mapric, Ipiranga, Brazil) and EDTA were weighed (RADWAG, Radom, Poland; AS 220/C/2) and added to a beaker to which distilled water was added and subjected to magnetic stirring (DiagTech, São Paulo, Brazil; DT3110H). Carpobol 940 was weighed, solubilized, and dispersed under magnetic stirring. It was neutralized with 50% triethanolamine (Dinâmica, Indaiatuba, Brazil) solution. The lyophilized *C. bracteosum* extract was dispersed in propylene glycol (Vetec, Rio de Janeiro, Brazil) (5%) to prepare gels containing 1% and 3% extract, respectively. The base gel was added to the extract and propylene glycol mixture.

### 2.6. Parameters of Carbopol-Based Gels Containing the C. bracteosum Extract

#### 2.6.1. pH and Conductivity

The pH was determined with a calibrated pH meter (IONLAB, PHS-3E, Araucária, Brazil), and the conductivity with a conductivity meter (Logen Scientific; Diadema, Brazil; D-300-K1) calibrated with a standard conductivity solution (146.9 μS/cm 25 °C, KCl 0.001 M). Both analyses were performed in triplicate for the carbopol base gel and the gels containing the extract. The pH and conductivity measurements were repeated for all samples on Days 7, 15, and 30. Dilutions were performed at a ratio of 1:10 in distilled water, according to the protocol of the Brazilian Pharmacopoeia, 6th edition [12].

#### 2.6.2. Rheology

The flow curves of the base gel and the gels containing 1% and 3% *C. bracteosum* extract were obtained using an Anton Paar rheometer (model MCR 92, Graz, Austria) with a Peltier cell for temperature control at 5 and 30 °C, using a cone and plate geometry (CP50-1 sensor) and a shear rate varying from 0.1 to 500 s^−1^.

#### 2.6.3. Spreadability

Sample spreadability was determined using the plate method. Briefly, each gel sample was applied to a glass plate mold with a central opening of 0.6 cm in diameter until it was filled. The mold plate was placed on a support plate, and after applying and leveling the sample, the mold plate was removed. Plate 1 (160 g) was placed on the sample, and the diameter of the spread gel was measured horizontally and vertically in millimeters to obtain the average diameter of the spread gel. The procedure was repeated with the addition of three more plates of increasing mass (227 g, 336 g, and 450 g, respectively). The spreadability, Si = spreadability, of the sample for a given weight i (mm^2^) was determined according to Equation (1):

Si = (d^2^ × π)/4 (1)

where d = average diameter (mm) and π = 3.14.

The procedure was performed in triplicate for all samples and repeated 30 days after the gels were prepared.

### 2.7. Extract and Gel Antibacterial Activity

The antibacterial activity of the *C. bracteosum* hydroethanolic extract and the gels were evaluated using the agar diffusion method [13] against 2 bacteria: *S. aureus* ATCC 25923 and *E. coli* ATCC 25922. Briefly, the inoculum was prepared by directly suspending colonies in phosphate-buffered saline (PBS) on a water plate for 24 h at 35 °C. Solution turbidity was adjusted according to the McFarland scale (bacterial density of 1 × 10^8^ CFU/mL). Twenty microlitre aliquots of the extract (10 mg/mL in 10% DMSO) and ten milligrams of the gel were added to sterile 6 mm discs and placed in Petri dishes containing culture medium with *S. aureus* and *E. coli* strains. Vancomycin (30 µg) and gentamicin (10 µg) were used as positive controls for *S. aureus* and *E. coli*, respectively.

## 3. Results and Discussion

### 3.1. Obtainment and Characterization of C. bracteosum Leaf Extract by UHPLC-MS/MS

The *C. bracteosum* hydroethanolic extract was obtained with a mass yield of 25%. Chemical profiling of the *C. bracteosum* extract revealed 18 compounds divided into 3 main secondary metabolite classes: organic acids, flavonoids, and biflavonoids. The list of annotated compounds and their corresponding chemical structures are presented in Table 1 and Figure 1, respectively. The chromatogram and MS^1^ and MS^2^ mass spectra of the annotated compounds are available in the Supplementary Materials (Figures S1–S19).

Secondary metabolites belonging to the phenolic compounds class have been reported in the literature for their antibacterial, antioxidant, and anti-inflammatory activities [14]. UHPLC-MS/MS analysis annotated five compounds from this class and others with acid characteristics: 5-methylnicotinic acid (**1**), quinic acid (**2**), dihydroferulic acid (**3**), xanthurenic acid (**4**), and ellagic acid (**11**). Among these metabolites, previous studies have highlighted the antibacterial activity of quinic acid against *Staphylococcus aureus*, including its ability to inhibit biofilm formation. The antibacterial effect of quinic acid is associated with disruption of the bacterial cell membrane and interference with cellular metabolic activity [15].

**Figure 1:**
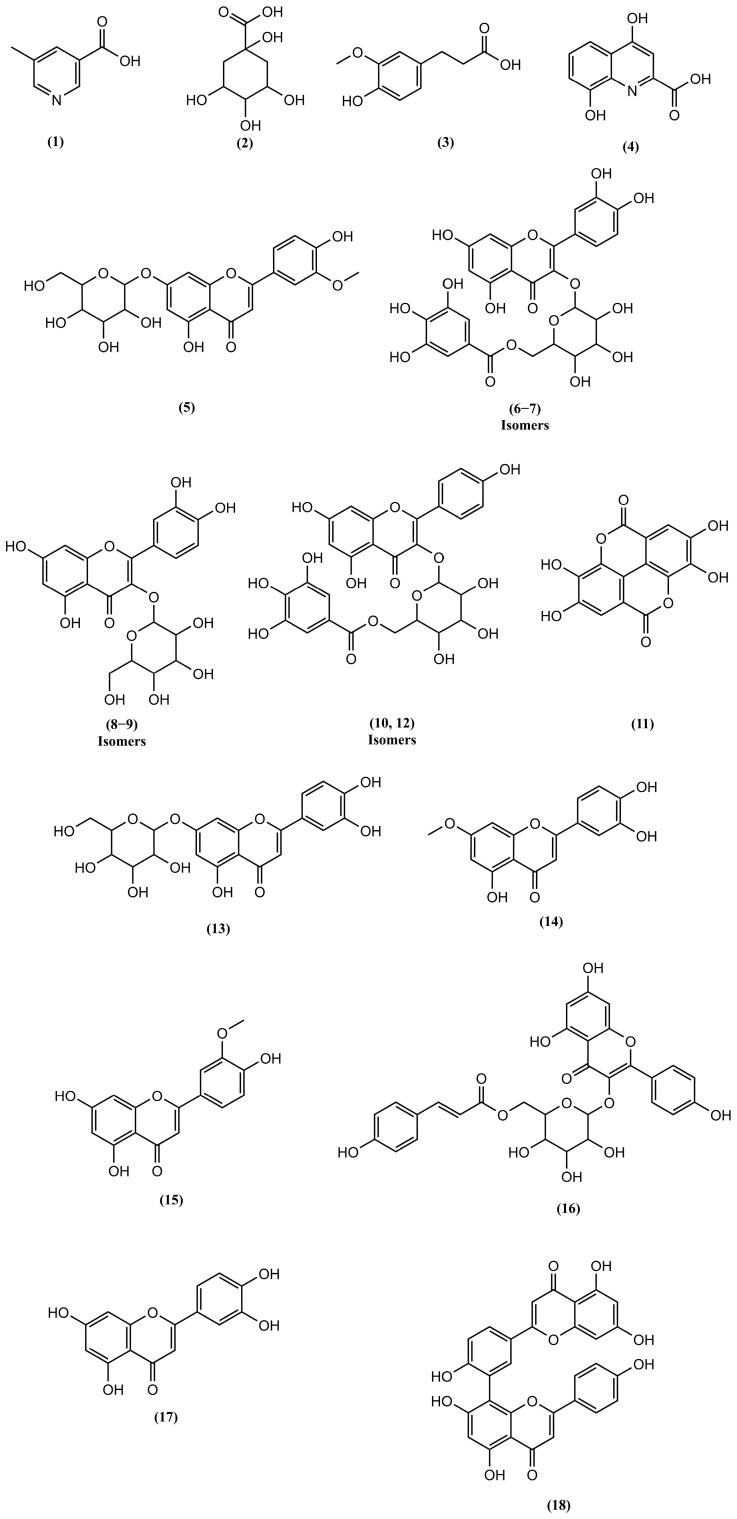
Chemical structure of molecules annotated in the *C. bracteosum* hydroethanolic extract during UHPLC-MS/MS analysis.

Twelve compounds belonging to the flavonoid class were also annotated (Figure 1, compounds **5**–**10** and **12**–**17**), occurring in either glycosylated or aglycone forms. The flavonoid class encompasses compounds belonging to two subclasses that are widely described in the literature for their antimicrobial, antioxidant, and anti-inflammatory properties [16,17]. Among these compounds, luteolin and glucoside derivatives of quercetin are known for their diverse bioactive properties [18,19]. In a study by Yu and colleagues [20], the activity of the glucosylated quercetin molecule was investigated, including quercetin 3-(6″-galloylglucoside), which is annotated herein. The results of the aforementioned study [20] demonstrated its efficacy against methicillin-resistant *Staphylococcus aureus* (MRSA), with a significant reduction in biofilm formation underscoring its therapeutic potential.

Amentoflavone (**18**) was also annotated in the hydroethanolic extract of *Cenostigma bracteosum*. This compound is a biflavonoid—a type of dimeric flavonoid composed of two linked flavonoid units—which has been widely reported in plant species of the *Cenostigma* genus, such as *C. pyramidalis* [4], in addition to its reported gastroprotective, anti-inflammatory, and antioxidant activities. A study by Hwang and colleagues [21] elucidated the antibacterial mechanism of the molecule. The testes indicated that amentoflavone induces hydroxyl radical generation through NADH depletion, a mechanism associated with the bacterial response to certain antibiotics [22]. This effect contributes to the antibacterial activity of amentoflavone and synergism with antibiotics. The synergism was showed in the hydroxyphenyl fluorescein test, which demonstrated a higher degree of hydroxyl radical formation in most cells treated with the combination of amentoflavone and ampicillin compared to those exposed to the antibiotics individually. *S. aureus* and *E. coli* were among the bacteria used in the test, thereby supporting the potential of amentoflavone to enhance antibiotic activity against these pathogens.

### 3.2. Cytotoxicity Assay of Cenostigma bracteosum Extract

**Figure 2:**
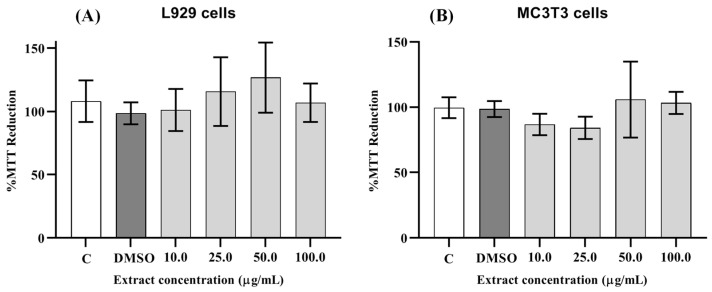
Effect of *Cenostigma bracteosum* hydroethanolic extract on cell viability—L929-CCL1 (**A**) and MC3T3-E1 (**B**) cells were cultured in the presence of extract (10–100 µg/mL) for 24 h. Group C—cells exposed only to the medium supplemented with 10% fetal bovine serum; Group DMSO—cells exposed to 1% DMSO. No statistically significant differences were observed between the values obtained from cells exposed to the samples and those from the control group (C). The *p*-value was 0.558 for L929 cells and 0.432 for MC3T3 cells.

Figure 2 shows the results of the cytotoxicity assay. The *C. bracteosum* extract exhibited no cytotoxic effects on L929 (A) and MC3T3 (B) cells at concentrations ranging from 10 to 100 µg/mL.

Cytotoxicity assays using a variety of available cell lines enable the rapid detection of toxic molecules in plant extracts with good sensitivity and reproducibility [23]. The L929 fibroblast cell line is widely utilized for evaluating the potential toxicity of substances based on their effects on cell viability and proliferation, owing to its high proliferative capacity. These fibroblasts are the most prevalent cells in all types of connective tissue and are involved in the synthesis and maintenance of the collagen-rich extracellular matrix. Therefore, assays employing this cell line are particularly suitable for assessing the cytotoxicity of biologically active compounds, including plant extracts [23]. There was no statistically significant difference between untreated and treated cells, regardless of the *C. bracteosum* extract concentration used, indicating low cytotoxicity toward murine dermal fibroblasts (L929). These results may be related to findings from extracts of other species within the Fabaceae family, such as ethanolic extracts from *Genista monspessulana* seeds, which exhibited anti-proliferative activity against cancer cell lines but were non-toxic to L929 cells at the same concentrations. These findings suggest that the *C. bracteosum* extract exhibits minimal toxicity toward normal fibroblastic cells [24].

A similar trend was observed in MC3T3-E1 pre-osteoblastic cells, where no cytotoxic effects were detected at concentrations up to 100 μg/mL of the extract. In this range, *C. bracteosum* extract proved to be less toxic than *Cissus quadrangularis* extracts and their hexane and dichloromethane fractions, which have been reported to reduce viability in MC3T3 cells at concentrations as low as 1 μg/mL [25]. These observations underscore the dose-dependent nature of cytotoxicity, highlighting the importance of concentration in determining whether a plant extract exerts therapeutic or harmful effects. For instance, the ethanolic extract of *Boesenbergia rotunda* not only exhibited no cytotoxicity but also promoted osteogenic differentiation and induced mineralization in MC3T3-E1 cells [26].

It was concluded that the hydroethanolic extract of *C. bracteosum* leaves is non-toxic in either L929 or MC3T3 cells at any of the concentrations tested. To our knowledge, this is the first report evaluating the cytotoxicity of *C. bracteosum* leaf extract. Future studies are warranted to determine the 50% inhibitory concentration (IC_50_) and further to investigate its cytotoxic profile across additional cell lines.

### 3.3. Obtainment of the C. bracteosum Gels

**Figure 3:**
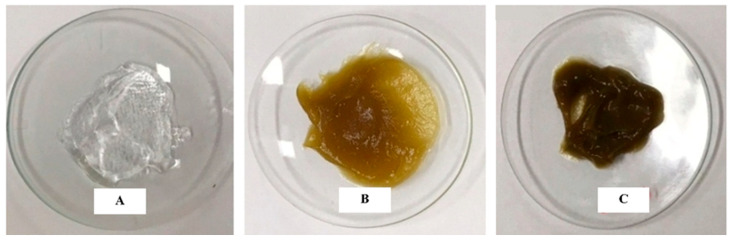
Carbopol-based gel/CBG (**A**), Cenostigma extract gel/CEG 1% (**B**), and Cenostigma extract gel/CEG 3% (**C**).

The carbopol-based gels were prepared to contain the hydroethanolic extract at 1% and 3% concentrations and the control group (base gel without extract). Figure 3 shows the carbopol-based gel (A), containing 1% (B) and 3% (C) of the incorporated extract.

### 3.4. Gel Characterization and Preliminary Stability

#### 3.4.1. pH and Conductivity

pH values of the carbopol-based gel and the 1%/3% extract gels are detailed in Table 2. There is a correlation between the amount of extract incorporated and the average pH. The pH was lower for the higher levels of incorporated extract due to the higher concentration of extract metabolites present in the gel matrix. No significant changes were observed in the pH values of gels with and without incorporated extract over the 30-day test period.

The difference in pH over time could be multifactorial, including environmental conditions such as gel formulation storage conditions pertaining to light and humidity exposure during the storage period. Therefore, storage time is one of the factors affecting gel pH stability, as phenomena such as metabolite hydrolysis or oxidation may occur in the incorporated extract [27]. Considering the pH values and the standard deviation of the gel sample containing 3% of the extract during the 30-day analysis, we can observe its compatibility with the skin’s pH (from 5.5 to 7.0), affording it more suitability for topical applications [28].

A direct correlation was also observed between the amount of extract incorporated into the gel and the increase in conductivity. In other words, the more extract added, the higher the conductivity (Table 3). This behavior is related to a higher concentration of ionizable polar compounds present in the extract, which increase the gel conductivity values when in solution [29]. Conductivity is an important characterization parameter associated with the rheology of the system. High conductivity is usually associated with coalescence phenomena, while very low values are associated with gel aggregation [30]. The base gel and the gels containing the *C. bracteosum* extract showed no visible instability over the 30-day evaluation period.

#### 3.4.2. Rheology

**Figure 4:**
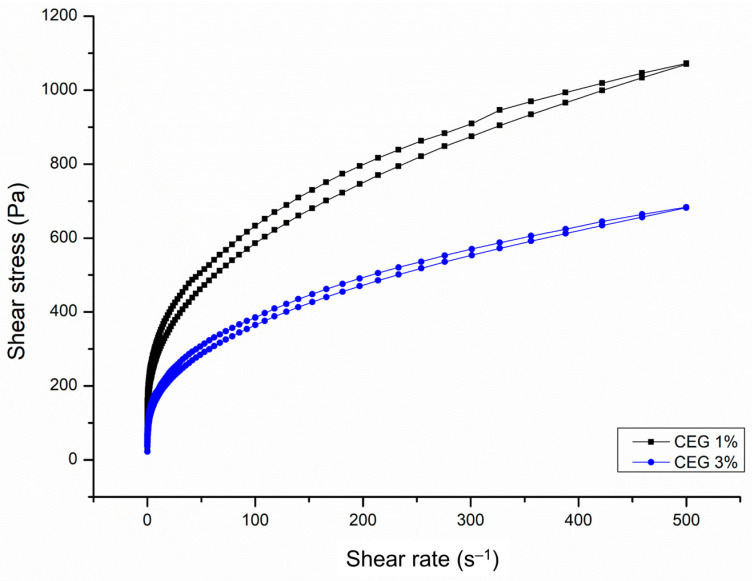
Flow curves of gels containing the *C. bracteosum* extract at 1% (CEG 1%—black) and 3% (CEG 3%—blue) concentrations.

The flow and viscosity curves of the gels containing the *C. bracteosum* hydroethanolic extract were obtained from the rheological analysis. Figure 4 shows the rheological behavior of the prepared gels in terms of shear stress expressed in Pa and shear rate expressed in units of s^−1^.

As shown in Figure 4, the gels produced with the *C. bracteosum* extract CEG 1% and CEG 3% exhibited non-Newtonian and pseudoplastic behavior, as the apparent viscosity decreased with increasing shear rate [31]. The rheological profile of both gels generated showed a thixotropic character, which, in practical terms, means that the gel becomes less viscous during topical application, making it easier to spread. After application, the initial viscosity is restored, thus preventing the gel from running on the skin [32]. This thixotropic behavior has been well documented and is expected for gels and hydrogels composed of polymers such as carbopol [33,34].

Lower viscosity was observed in the CEG 3% samples than the CEG 1%, and the flow curves indicated a lower thixotropic character in the CEG 3% gel. The higher content of added extract is associated with a greater availability of molecules interacting with the polymeric matrix of the gel, and a change in pH, results in variations in the nature and intensity of these interactions, namely reduced cross-linking and consequently lower viscosity [35]. In the CEG 3% gel, a slight decrease in viscosity values was observed after the 30-day storage period, with its thixotropic behavior maintained, as evidenced by the maintenance of the hysteresis area in the flow curve. In the literature, it is common to observe a decrease in gel viscosity in stability tests due to breakages in the polymer chain used to form the gel [27].

Gel viscosity is closely related to adhesion, which may affect its therapeutic effect. The longer the gel adheres to the skin, the more the active ingredients are absorbed, resulting in a more prolonged therapeutic effect and increased effectiveness [36]. Therefore, it was a positive and desirable result that the gels containing the *C. bracteosum* extract maintained similar viscosity values for 30 days after preparation.

#### 3.4.3. Spreadability

**Figure 5:**
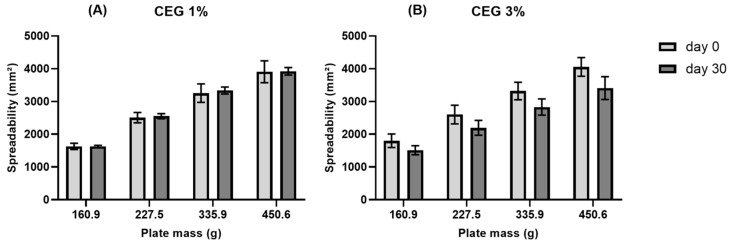
Spreadability of CEG 1% (**A**) and CEG 3% (**B**) gels on Days 0 and 30.

Spreadability is an important characterization parameter for topical formulations. Gels with higher spreadability produce thinner films and are highly recommended [37]. Figure 5 shows that the CEG 1% gel did not exhibit any significant change in the spreadability values after the 30-day storage period. Conversely, the CEG 3% gel presented a decrease in viscosity (*p* < 0.05) after the application of the plate with the highest mass (450.6 g).

In another study, carbopol 940 was used in 1%, 1.5%, 2%, and 2.5% proportions to incorporate a 4% ethanolic extract of *Pothos scandens* L. The highest spreadability value was observed in the gel prepared with 1% carbopol 940 [38], the same proportion of the polymer used to form gels with the *C. bracteosum* extract in this study. Despite the slight reduction in spreadability of the CEG 3% gel, the appearance and texture of the gel remained suitable for topical application.

### 3.5. Antibacterial Activity of the C. bracteosum Extract and Gel

The antibacterial activities of the *C. bracteosum* hydroethanolic leaves extract, together with the CBG, CEG 1%, and CEG 3% gels, were evaluated against *S. aureus* and *E. coli* strains using the diffusion technique, measuring the halo of inhibition formed (Table 4 and Figure S20). Vancomycin (*S. aureus*) and gentamicin (*E. coli*) were used as antibiotic-positive controls. The inhibition halos formed for the *C. bracteosum* extract were 15.5 ± 0.7 mm and 11.5 ± 0.7 mm for *S. aureus* and *E. coli*, respectively.

Only one study reported the antibacterial activity of a methanolic extract of the leaves of this species against *Staphylococcus saprophyticus* and *Pseudomonas aeruginosa* strains [3]. The antibacterial activity observed in the *C. bracteosum* hydroethanolic extract may be related to the presence of the secondary metabolites annotated in the UHPLC-MS/MS analysis. For example, phenolic acids have already demonstrated antibacterial activity [14]. Flavonoids can break down the plasma membrane and bacterial walls, interrupting nucleic acid synthesis and energy metabolism [3]. Biflavonoids, involving the amentoflavone mechanism, induce the formation of these radicals inside the bacteria by depleting NADH, resulting in oxidative stress and, consequently, bacterial death [21].

Regarding the gels, only CEG 3% inhibited *S. aureus* with an inhibition halo of 6.0 mm. For comparison, the inhibition halo of vancomycin (30 µg) on the same plate was 19 mm. Since the mass of gel on the surface of the plate was 10 mg, the effective doses of the *C. bracteosum* extract were 100 and 300 µg for the CEG 1% and CEG 3% gels, respectively. No inhibitory effects were observed for the gels against *E. coli* at the aforementioned levels of incorporated extract. These results open new perspectives for using this formulation with other antibacterial test methods to calculate the minimum inhibitory concentration, tests with other bacteria, and even other *in vitro* activities and *in vivo* confirmations.

## 4. Conclusions

The hydroethanolic extract of *C. bracteosum* leaves demonstrated antibacterial activity against *S. aureus* and *E. coli*. Additionally, the extract showed no cytotoxicity toward L929 fibroblasts and MC3T3 pre-osteoblast cells at the maximum tested concentration (100 µg/mL). UHPLC-MS/MS analysis enabled the annotation of 18 secondary metabolites, primarily phenolic compounds and other acidic compounds, including 5-methylnicotinic acid, flavonoids, and biflavonoids. The gels prepared showed satisfactory stability results 30 days post-production. Despite the increased conductivity values, no phenomena indicative of physicochemical instability was observed. Both viscosity and spreadability exhibited the expected behavior, with an effective correlation between these parameters. From the pH analysis, it could be concluded that Cenostigma extract gel (CEG) 3% was the most adapted to the skin pH range and the formulation that inhibited *S. aureus* growth. The results presented herein constitute a formal report on the chemical diversity of the secondary metabolites of a Caatinga biome species, underscoring the need for further studies involving this common plant in the development of gel applications.

## Figures and Tables

**Table 1:** Compounds annotated in the *C. bracteosum* hydroethanolic extract by UHPLC-MS/MS analysis.

Peak	Compound	Retention Time (min)	Molecular Formula	*m*/*z* Theoretical [M + H]^+^	*m*/*z* Experimental [M + H]^+^	Error (ppm)	Fragments (*m*/*z*)
**1**	5-methylnicotinic acid	1.81	C_7_H_7_NO_2_	138.0550	138.0545	−3.6	92.
**2**	Quinic acid	1.89	C_7_H_12_O_6_	193.0707	193.0711	2.0	157, 147, 139, 129, 121, 111.
**3**	Dihydroferulic acid	2.50	C_10_H_12_O_4_	197.0808	197.0797	−5.6	147, 137, 119.
**4**	Xanthurenic acid	5.30	C_10_H_7_NO_4_	206.0448	206.0455	3.4	188, 178, 160, 132, 105, 77.
**5**	Chrysoeriol 7-*O*-glucoside	6.98	C_22_H_22_O_11_	463.1235	463.1247	2.6	301, 286.
**6**	Quercetin 3-(6″-galloylglucoside) isomer	7.97	C_28_H_24_O_16_	617.1137	617.1128	−1.4	315, 303, 153.
**7**	Quercetin 3-(6″-galloylglucoside) isomer	8.05	C_28_H_24_O_16_	617.1137	617.1137	0	315, 303, 153.
**8**	Isoquercetin isomer	8.28	C_21_H_20_O_12_	465.1028	465.1028	0	303.
**9**	Isoquercetin isomer	8.36	C_21_H_20_O_12_	465.1028	465.1045	3.6	303.
**10**	Kaempferol 3-(6″-galloylglucoside) isomer	8.43	C_28_H_24_O_15_	601.1188	601.1203	2.5	315, 287, 153.
**11**	Ellagic acid	8.59	C_14_H_6_O_8_	303.0135	303.0135	0	303, 229, 201.
**12**	Kaempferol 3-(6″-galloylglucoside) isomer	8.74	C_28_H_24_O_15_	601.1188	601.1180	−1.3	315, 287, 153.
**13**	Luteolin 7-*O*-glucoside	8.89	C_21_H_20_O_11_	449.1078	449.1083	1.1	287.
**14**	7-*O*-Methyl luteolin	9.04	C_16_H_12_O_6_	301.0707	301.0708	0.3	286, 258.
**15**	Chrysoeriol	9.12	C_16_H_12_O_6_	301.0707	301.0707	0	286, 258.
**16**	Kaempferol-3-glucoside-6″-*p*-coumaroyl	10.71	C_30_H_26_O_13_	595.1446	595.1492	7.7	309, 287, 147.
**17**	Luteolin	11.18	C_15_H_10_O_6_	287.0550	287.0547	−1.0	201, 153, 135.
**18**	Amentoflavone	15.51	C_30_H_18_O_10_	539.0973	539.0979	1.1	497, 431, 387.

**Table 2:** pH values of the gels: carbopol-based gel (CBG), Cenostigma extract gel 1% (CEG 1%), and Cenostigma extract gel 3% (CEG 3%) over 30 days.

Samples (pH ± sd ^1^)	D0	D7	D15	D30
CBG	7.9 ± 0.11	8.0 ± 0.07	8.0 ± 0.04	7.9 ± 0.03
CEG 1%	6.8 ± 0.43	7.1 ± 0.03	7.1 ± 0.08	7.2 ± 0.12
CEG 3%	6.7 ± 0.08	6.5 ± 0.07	6.4 ± 0.11	6.3 ± 0.14

^1^ sd: standard deviation. D = day.

**Table 3:** Gel conductivity values: carbopol-based gel (CBG), Cenostigma extract gel 1% (CEG 1%), and Cenostigma extract gel 3% (CEG 3%) over 30 days.

Samples (µS/cm ± sd ^1^)	D0	D7	D15	D30
CBG	280.4 ± 36.1	333.2 ± 23.0	351.4 ± 34.2	459.9 ± 71.3
CEG 1%	346.9 ± 51.8	544.0 ± 96.8	660.3 ± 72.8	495.8 ± 28.1
CEG 3%	484.5 ± 7.9	674.3 ± 19.6	733.0 ± 70.1	825.1 ± 79.2

^1^ sd: standard deviation. D = day.

**Table 4:** Comparison of inhibition halo size (mm) against *S. aureus* and *E. coli* formed around the *C. bracteosum* extract, CBG (carbopol based-gel), CEG 1% (Cenostigma extract gel 1%), CEG 3% (Cenostigma extract gel 3%), and controls (vancomycin and gentamicin).

Samples	*S. aureus* (mm ± sd ^1^)	*E. coli* (mm ± sd ^1^)
*C. bracteosum* extract	15.5 ± 0.7	11.5 ± 0.7
CBG	0.0	0.0
CEG 1%	0.0	0.0
CEG 3%	6.0 ± 0.0	0.0
Vancomycin (30 µg)	20 ± 0.6	-
Gentamicin (10 µg)	-	19 ± 0.6

^1^ sd: standard deviation.

## Data Availability

The data from GNPS analysis will be made available by the corresponding author request.

## Supplementary Materials

The following supporting information can be downloaded at https://www.mdpi.com/article/10.3390/pharmaceutics17060780/s1, Figure S1: Chromatogram of *C. bracteosum* hydroethanolic extract by UHPLC-MS/MS analysis. Figure S2: Mass spectra (MS^1^ and MS^2^) and UV-VIS spectra of 5-methylnicotinic acid, one of the substances annotated in the hydroethanolic extract of *C. bracteosum*. Figure S3: Mass spectra (MS^1^ and MS^2^) and UV-VIS spectra of Quinic Acid, one of the substances annotated in the hydroethanolic extract of *C. bracteosum*. Figure S4: Mass spectra (MS^1^ and MS^2^) and UV-VIS spectra of dihydroferulic acid, one of the substances annotated in the hydroethanolic extract of *C. bracteosum*. Figure S5: Mass spectra (MS^1^ and MS^2^) and UV-VIS spectra of xanthurenic acid, one of the substances annotated in the hydroethanolic extract of *C. bracteosum*. Figure S6: Mass spectra (MS^1^ and MS^2^) and UV-VIS spectra of Chrysoeriol 7-*O*-glucoside, one of the substances annotated in the hydroethanolic extract of *C. bracteosum*. Figure S7: Mass spectra (MS^1^ and MS^2^) and UV-VIS spectra of quercetin 3-(6″-galloylglucoside) isomer, one of the substances annotated in the hydroethanolic extract of *C. bracteosum*. Figure S8: Mass spectra (MS^1^ and MS^2^) and UV-VIS spectra of quercetin 3-(6″-galloylglucoside) isomer, one of the substances annotated in the hydroethanolic extract of *C. bracteosum*. Figure S9: Mass spectra (MS^1^ and MS^2^) and UV-VIS spectra of isoquercetin isomer, one of the substances annotated in the hydroethanolic extract of *C. bracteosum*. Figure S10: Mass spectra (MS^1^ and MS^2^) and UV-VIS spectra of isoquercetin isomer, one of the substances annotated in the hydroethanolic extract of *C. bracteosum*. Figure S11: Mass spectra (MS^1^ and MS^2^) and UV-VIS spectra of Kaempferol 3-(6″-galloylglucoside) isomer, one of the substances annotated in the hydroethanolic extract of *C. bracteosum*. Figure S12: Mass spectra (MS^1^ and MS^2^) and UV-VIS spectra of ellagic acid, one of the substances annotated in the hydroethanolic extract of *C. bracteosum*. Figure S13: Mass spectra (MS^1^ and MS^2^) and UV-VIS spectra of Kaempferol 3-(6″-galloylglucoside) isomer, one of the substances annotated in the hydroethanolic extract of *C. bracteosum*. Figure S14: Mass spectra (MS^1^ and MS^2^) and UV-VIS spectra of Luteolin 7-*O*-glucoside, one of the substances annotated in the hydroethanolic extract of *C. bracteosum*. Figure S15: Mass spectra (MS^1^ and MS^2^) and UV-VIS spectra of 7-*O*-Methyl luteolin, one of the substances annotated in the hydroethanolic extract of *C. bracteosum*. Figure S16: Mass spectra (MS^1^ and MS^2^) and UV-VIS spectra of Chrysoeriol, one of the substances annotated in the hydroethanolic extract of *C. bracteosum*. Figure S17: Mass spectra (MS^1^ and MS^2^) and UV-VIS spectra of Kaempferol-3-glucoside-6″-*p*-coumaroyl, one of the substances annotated in the hydroethanolic extract of *C. bracteosum*. Figure S18: Mass spectra (MS^1^ and MS^2^) and UV-VIS spectra of luteolin, one of the substances annotated in the hydroethanolic extract of *C. bracteosum*. Figure S19: Mass spectra (MS^1^ and MS^2^) and UV-VIS spectra of amentoflavone, one of the substances annotated in the hydroethanolic extract of *C. bracteosum*. Figure S20: Inhibition halos formed in samples of *C. bracteosum* gels on *S. aureus* and *E. coli*. Sample codes follow the same convention as in the main manuscript: CBG—carbopol-based gel without extract; CEG 1%—gel containing 1% *C. bracteosum* extract; CEG 3%—gel containing 3% *C. bracteosum* extract. An inhibition zone was observed only for the CEG 3% sample (left side of the image).

## Abbreviations

The following abbreviations are used in this manuscript:

ATCC	American Type Culture Collection
UHPLC-MS/MS	Ultra High-Performance Liquid Chromatography–Mass Spectrometry/Mass Spectrometry
FLONA	Assu National Forest
SisGen	Genomic Management System
SISBIO	Authorization and Information System for Biodiversity
DAD	Diode Array Detector
Q-TOF	Quadrupole-Time of Flight mass spectrometer
ESI	Electrospray Ionization
MS	Mass Spectrum
GNPS2	Global Natural Products Social Molecular Networking 2
EDTA	Ethylenediamine Tetraacetic Acid
PBS	Phosphate-Buffered Saline
DMSO	Dimethyl Sulfoxide
MRSA	Methicillin-Resistant *Staphylococcus aureus*
NADH	Nicotinamide Adenine Dinucleotide reduced form
CBG	Carbopol-Based Gel
CEG 1%	Carbopol-Based Gel containing 1% *C. bracteosum* extract
CEG 3%	Carbopol-Based Gel containing 3% *C. bracteosum* extract
