# Supplementary material for: Cenostigma bracteosum Hydroethanolic Extract: Chemical Profile, Antibacterial Activity, Cytotoxicity, and Gel Formulation Development
Source: Pharmaceutics. 2025 Jun 14;17(6):780. doi: 10.3390/pharmaceutics17060780 (PMC12196441; doi:10.3390/pharmaceutics17060780)
Supplement: Supplementary file 1 [file pharmaceutics-17-00780-s001.zip › pharmaceutics-3708185-supplementary.pdf]

Supplementary material

Cenostigma bracteosum Hydroethanolic Extract: Chemical Profile, Antibacterial Activity, Cytotoxicity, and Gel Formulation Development

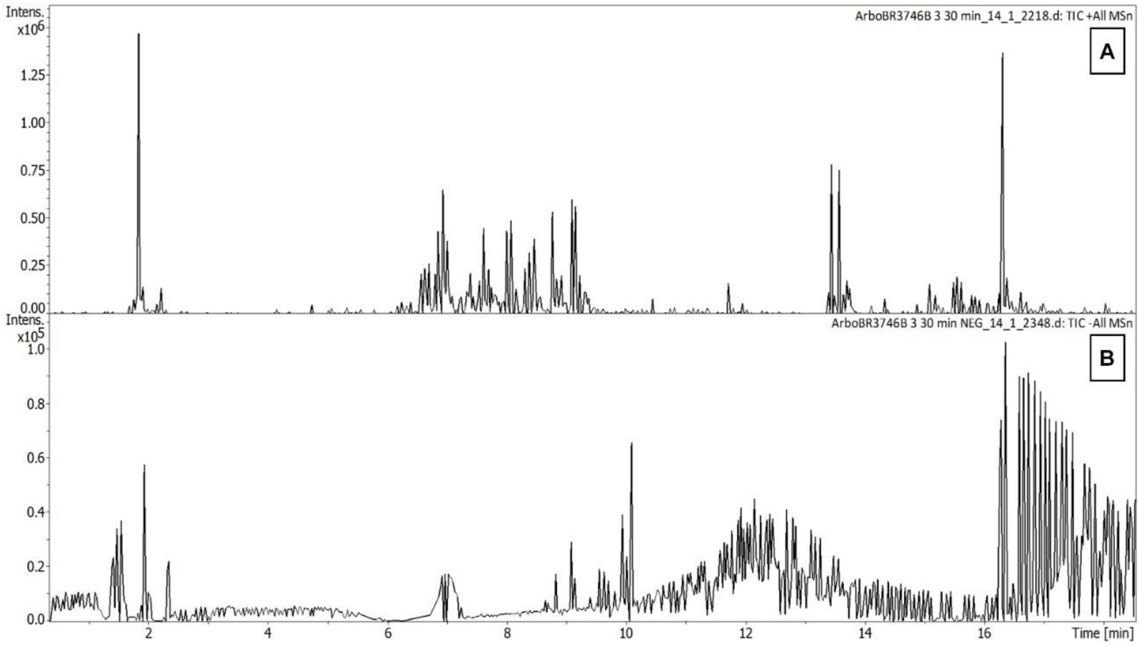

Figure S1. Chromatogram of *C. bracteosum* hydroethanolic extract by UHPLC-MS/MS analysis

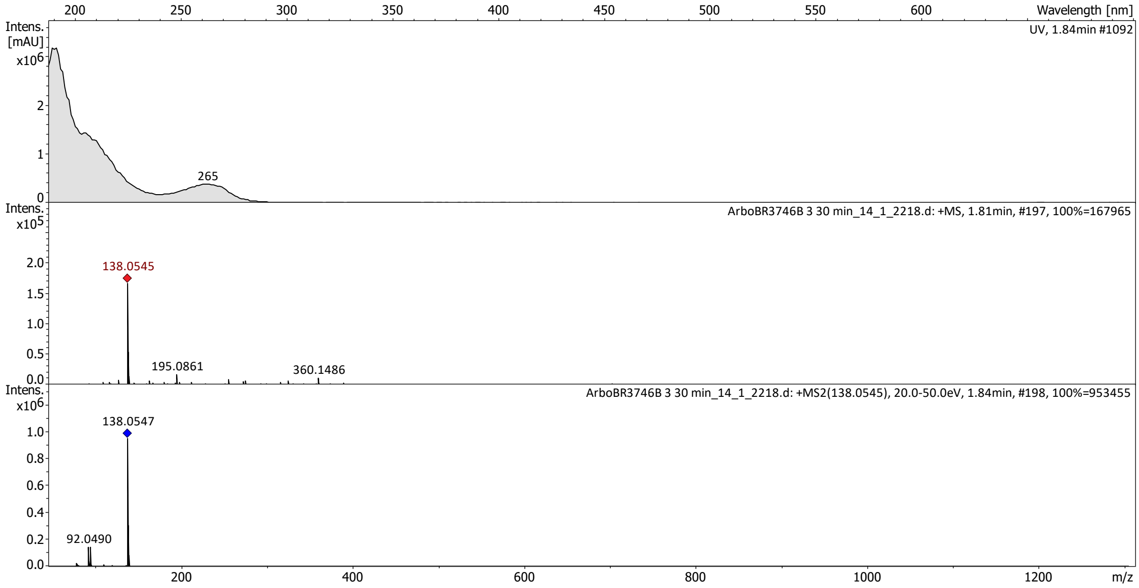

Figure S2. Mass spectra (MS^1 and MS^2) and UV-VIS spectra of 5-Methylnicotinic Acid, one of the substances annotated in the hydroethanolic extract of *C. bracteosum*.

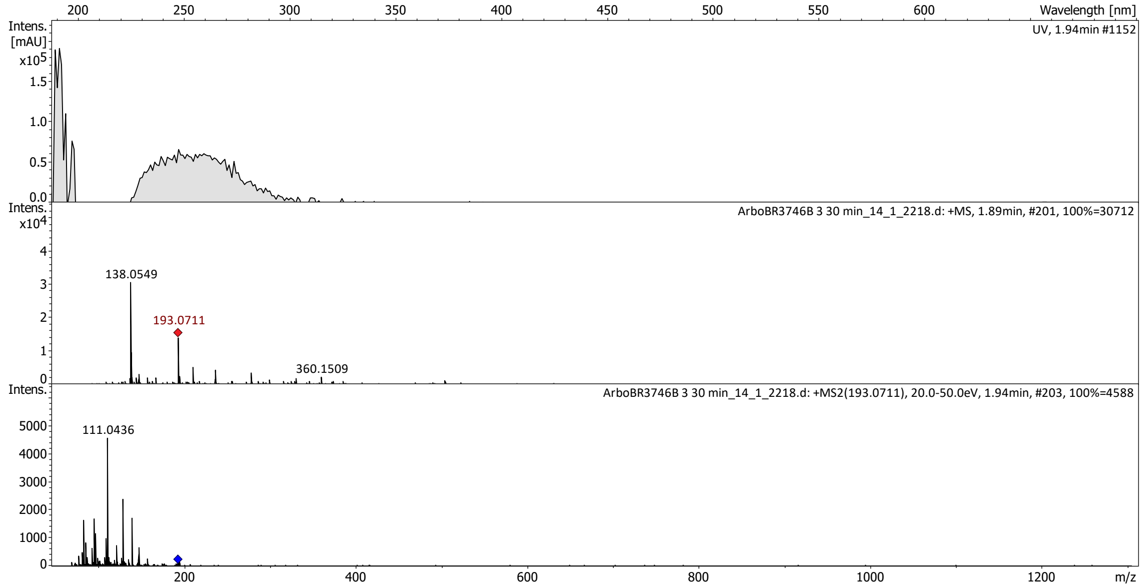

Figure S3. Mass spectra (MS[1] and MS[2]) and UV-VIS spectra of Quinic Acid, one of the substances annotated in the hydroethanolic extract of *C. bracteosum*.

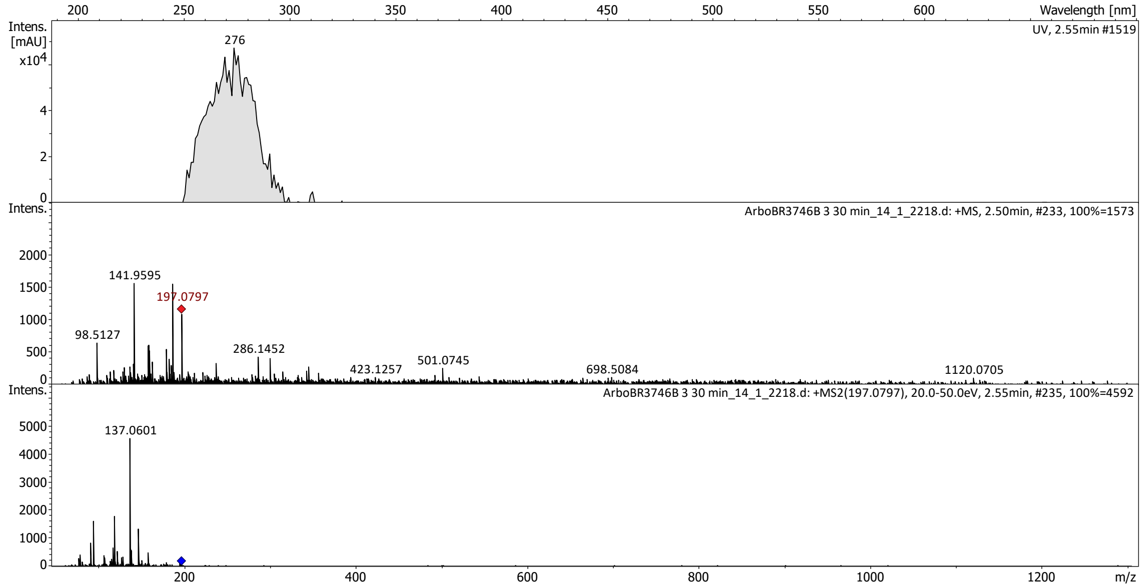

Figure S4. Mass spectra (MS[1] and MS[2]) and UV-VIS spectra of Dihydroferulic Acid, one of the substances annotated in the hydroethanolic extract of *C. bracteosum*.

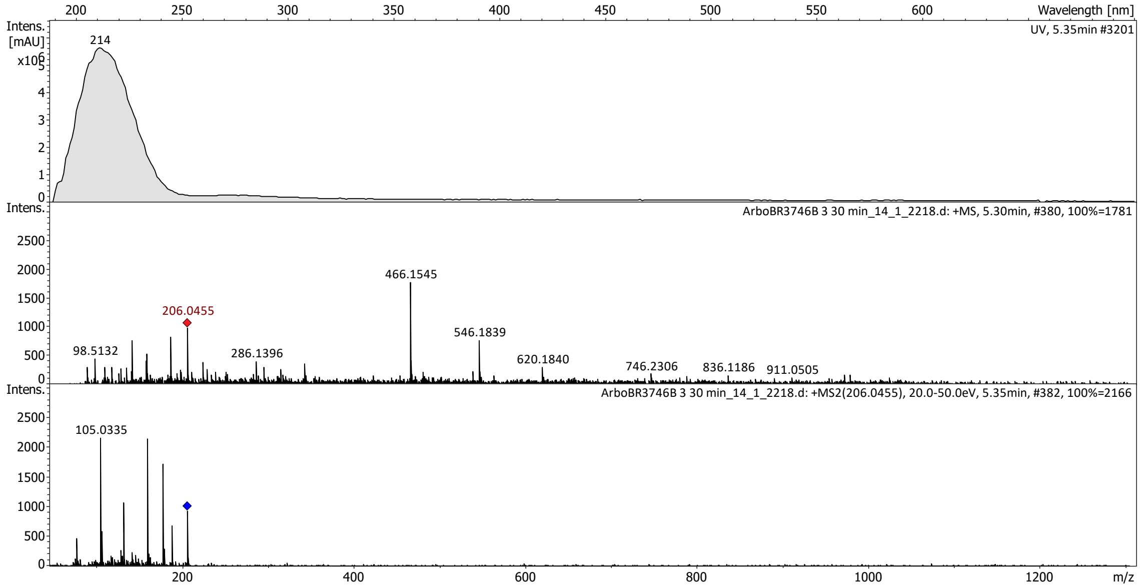

Figure S5. Mass spectra (MS^1 and MS^2) and UV-VIS spectra of Xanthurenic Acid, one of the substances annotated in the hydroethanolic extract of *C. bracteosum*.

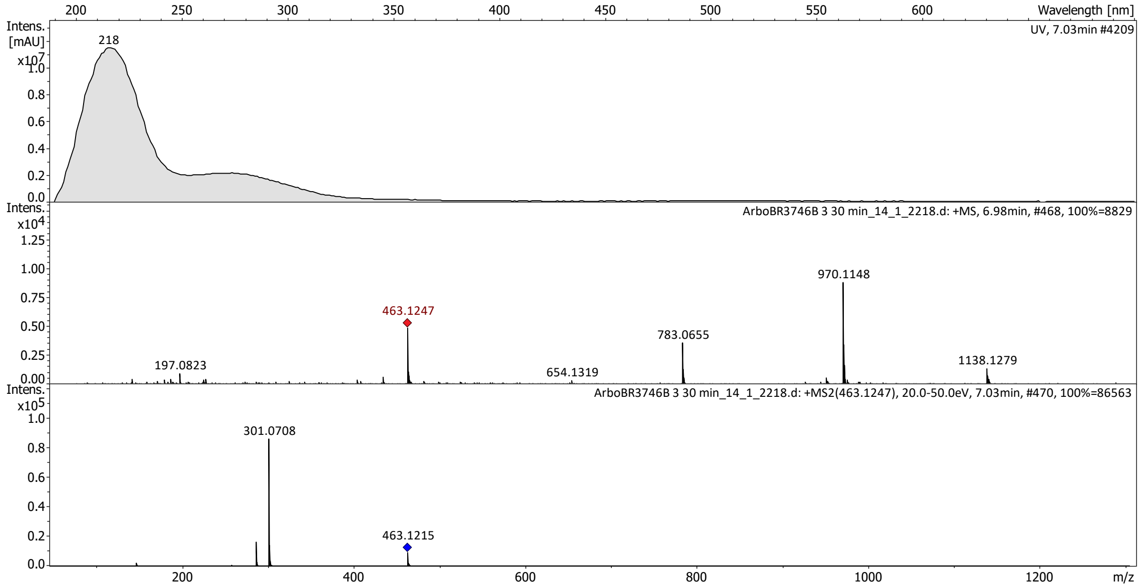

Figure S6. Mass spectra (MS^1 and MS^2) and UV-VIS spectra of Chrysoeriol 7-*O*-glucoside, one of the substances annotated in the hydroethanolic extract of *C. bracteosum*.

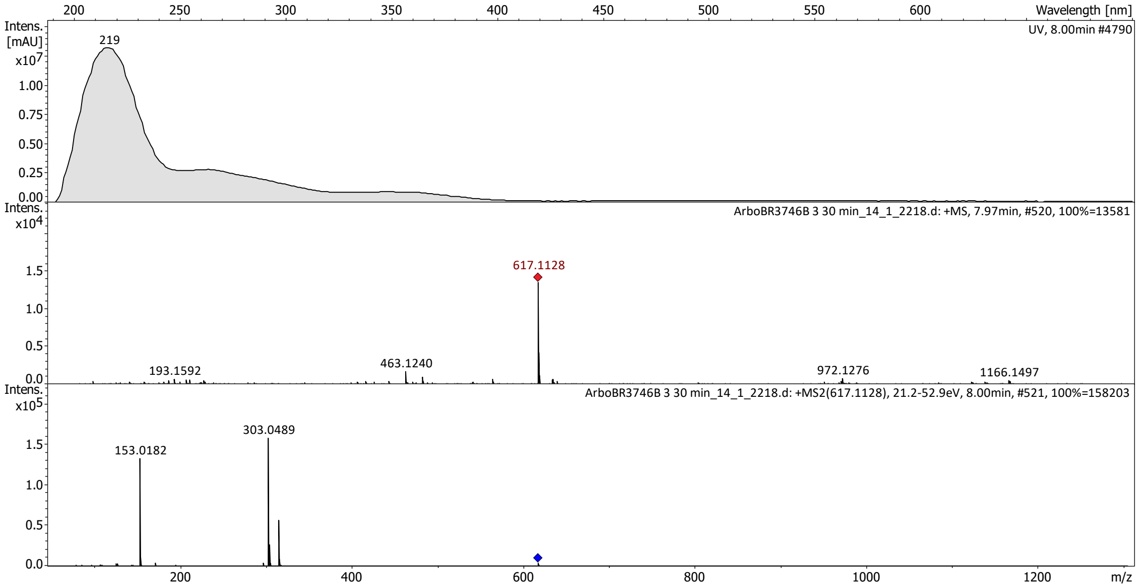

Figure S7. Mass spectra (MS^1 and MS^2) and UV-VIS spectra of Quercetin 3-(6''-galloylglucoside) isomer, one of the substances annotated in the hydroethanolic extract of *C. bracteosum*.

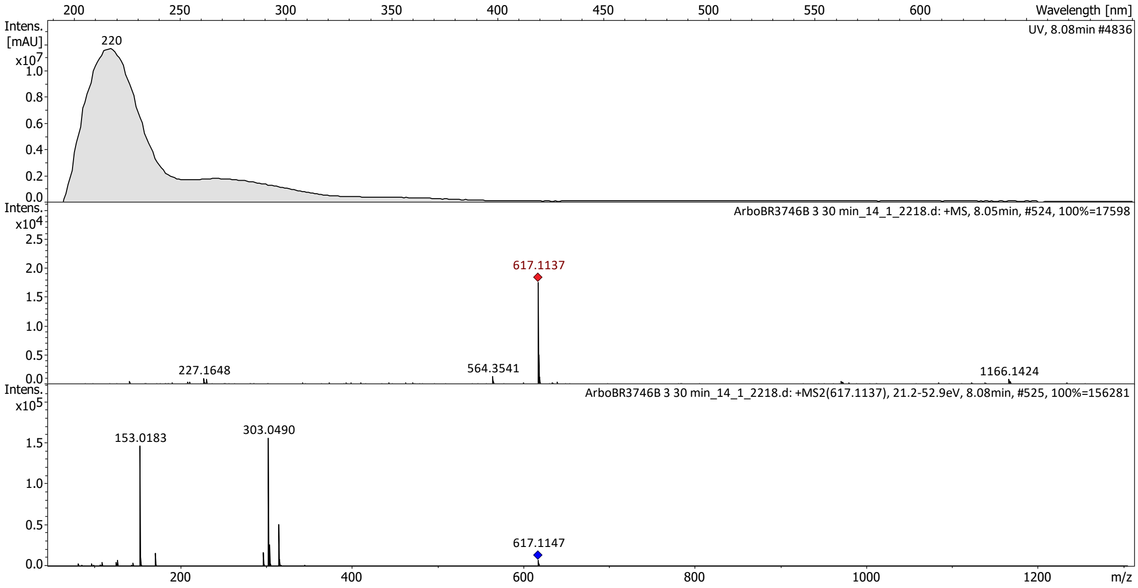

Figure S8. Mass spectra (MS^1 and MS^2) and UV-VIS spectra of Quercetin 3-(6''-galloylglucoside) isomer, one of the substances annotated in the hydroethanolic extract of *C. bracteosum*.

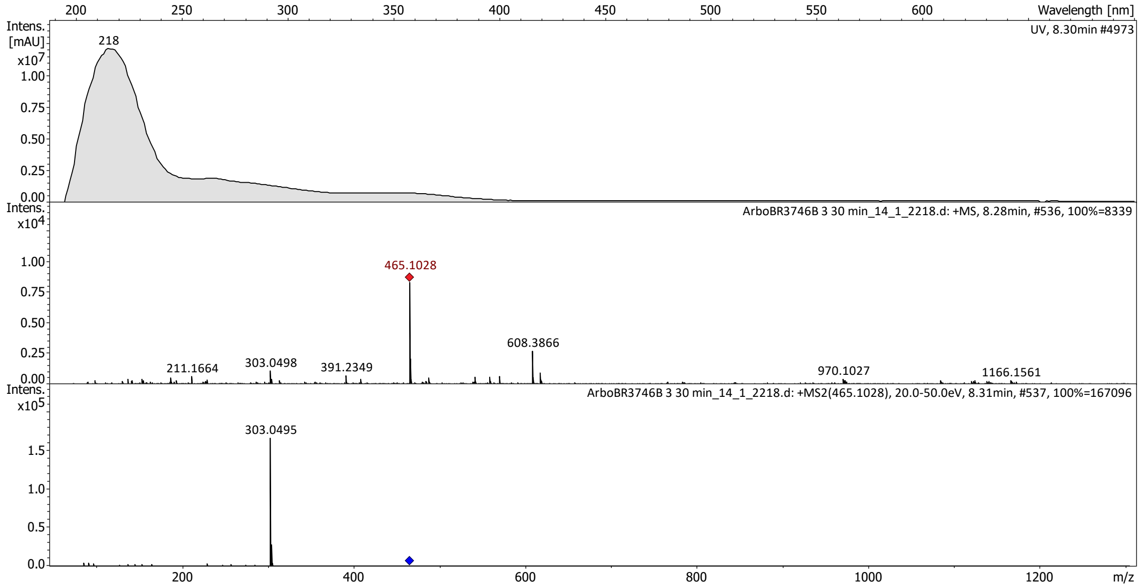

Figure S9. Mass spectra (MS[1] and MS[2]) and UV-VIS spectra of Isoquercetin isomer, one of the substances annotated in the hydroethanolic extract of *C. bracteosum*.

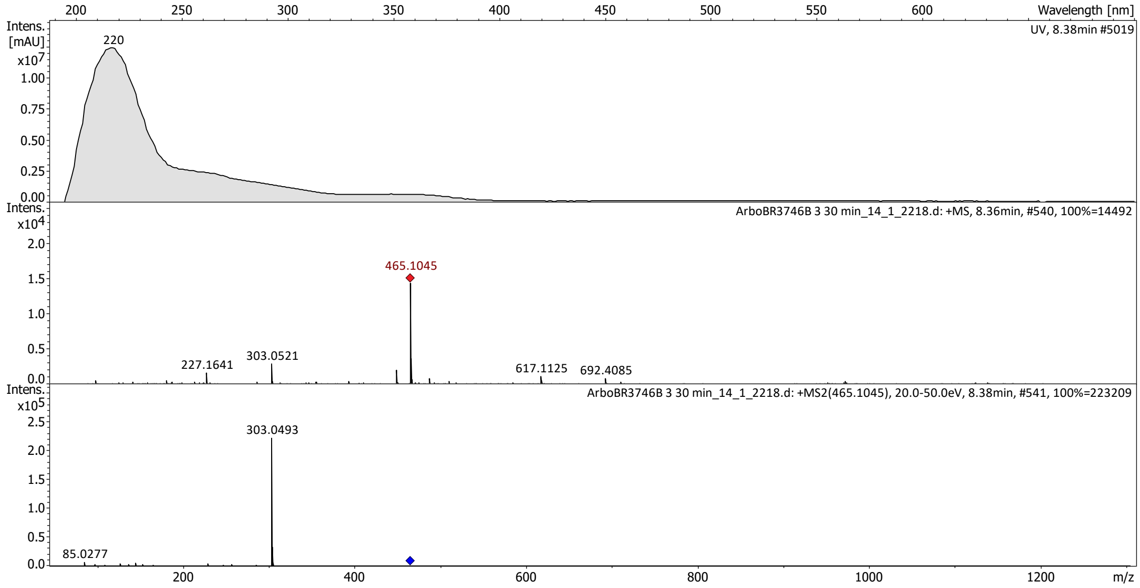

Figure S10. Mass spectra (MS[1] and MS[2]) and UV-VIS spectra of Isoquercetin isomer one, of the substances annotated in the hydroethanolic extract of *C. bracteosum*.

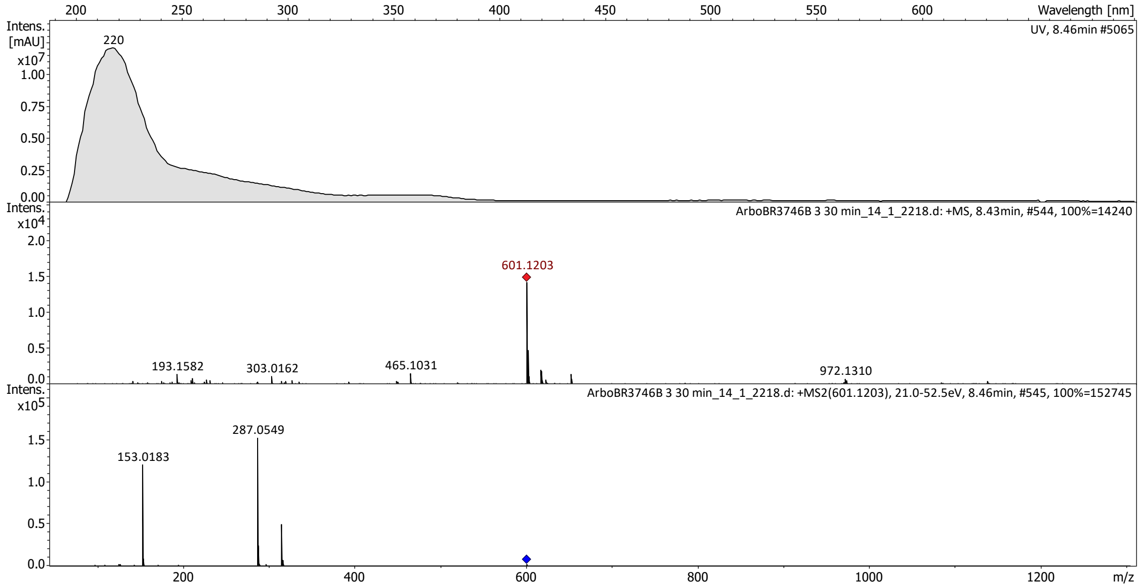

Figure S11. Mass spectra (MS[1] and MS[2]) and UV-VIS spectra of Kaempferol 3-(6''-galloylglucoside) isomer, one of the substances annotated in the hydroethanolic extract of *C. bracteosum*.

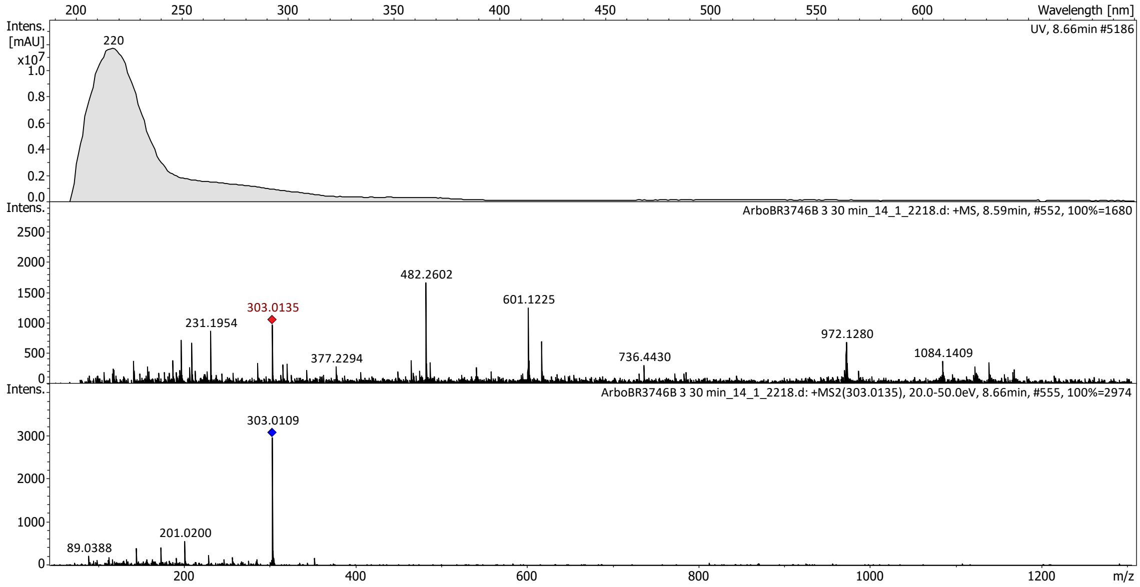

Figure S12. Mass spectra (MS[1] and MS[2]) and UV-VIS spectra of Ellagic acid, one of the substances annotated in the hydroethanolic extract of *C. bracteosum*.

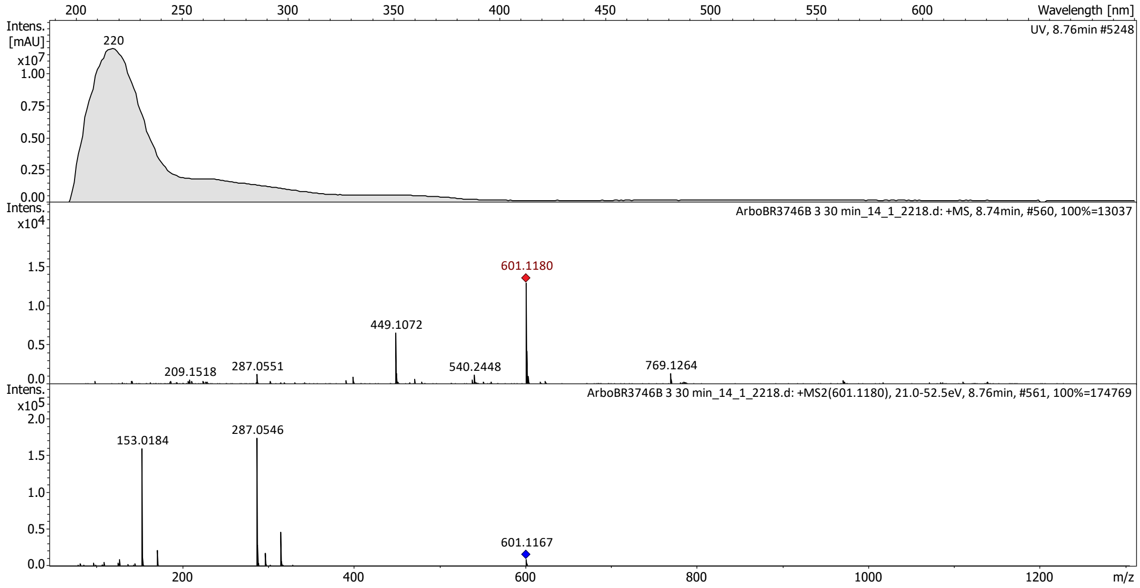

Figure S13. Mass spectra (MS^1 and MS^2) and UV-VIS spectra of Kaempferol 3-(6''-galloylglucoside) isomer, one of the substances annotated in the hydroethanolic extract of *C. bracteosum*.

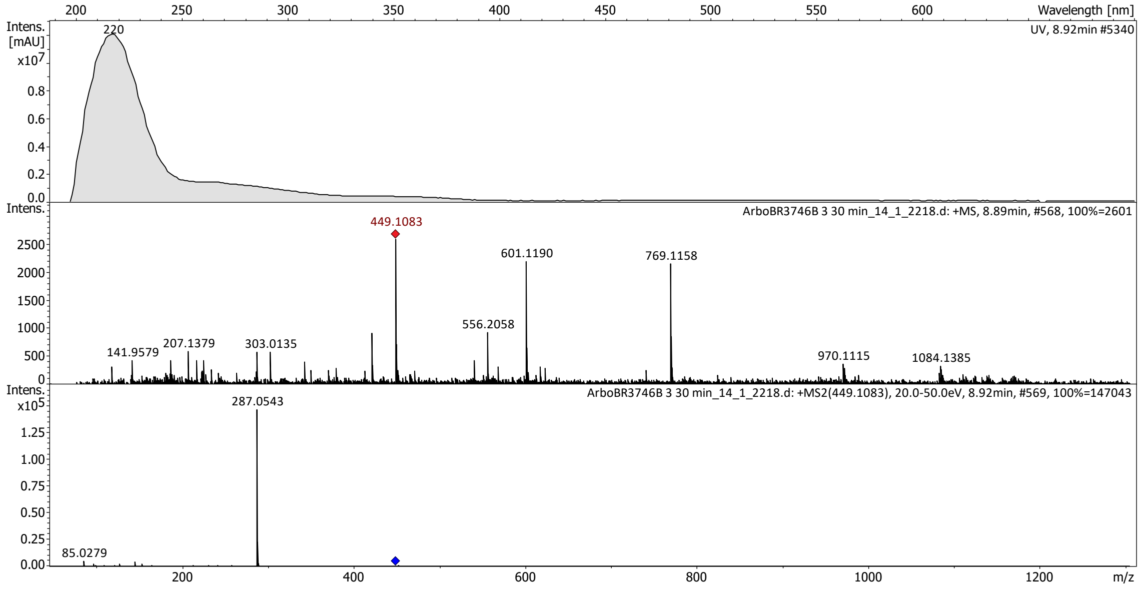

Figure S14. Mass spectra (MS^1 and MS^2) and UV-VIS spectra of Luteolin 7-*O*-glucoside, one of the substances annotated in the hydroethanolic extract of *C. bracteosum*.

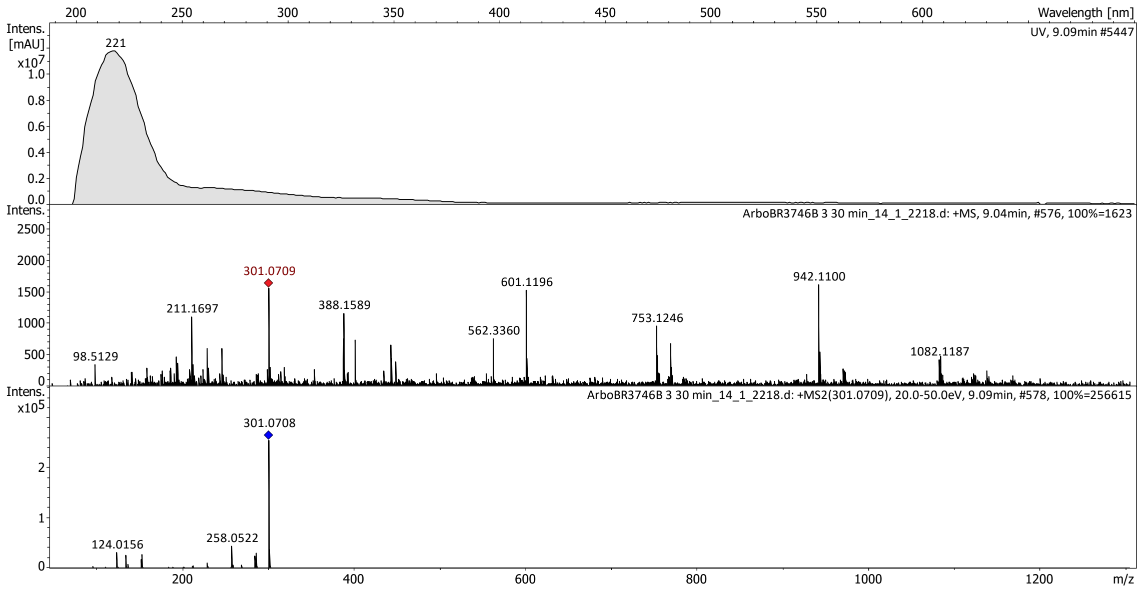

Figure S15. Mass spectra (MS[1] and MS[2]) and UV-VIS spectra of 7-*O*-Methyl luteolin, one of the substances annotated in the hydroethanolic extract of *C. bracteosum*.

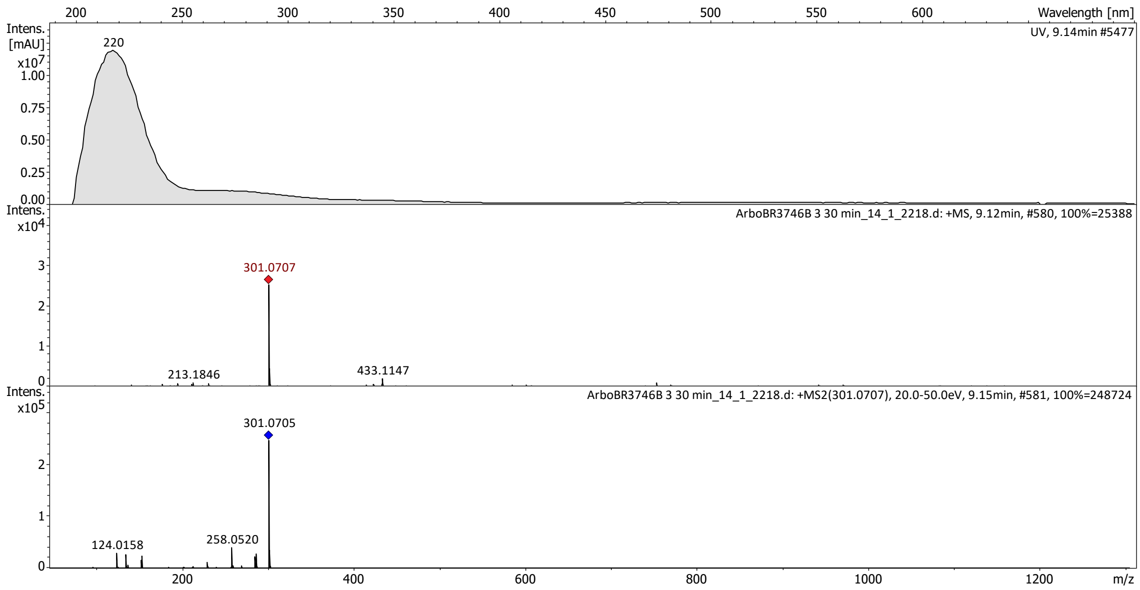

Figure S16. Mass spectra (MS[1] and MS[2]) and UV-VIS spectra of Chrysoeriol, one of the substances annotated in the hydroethanolic extract of *C. bracteosum*.

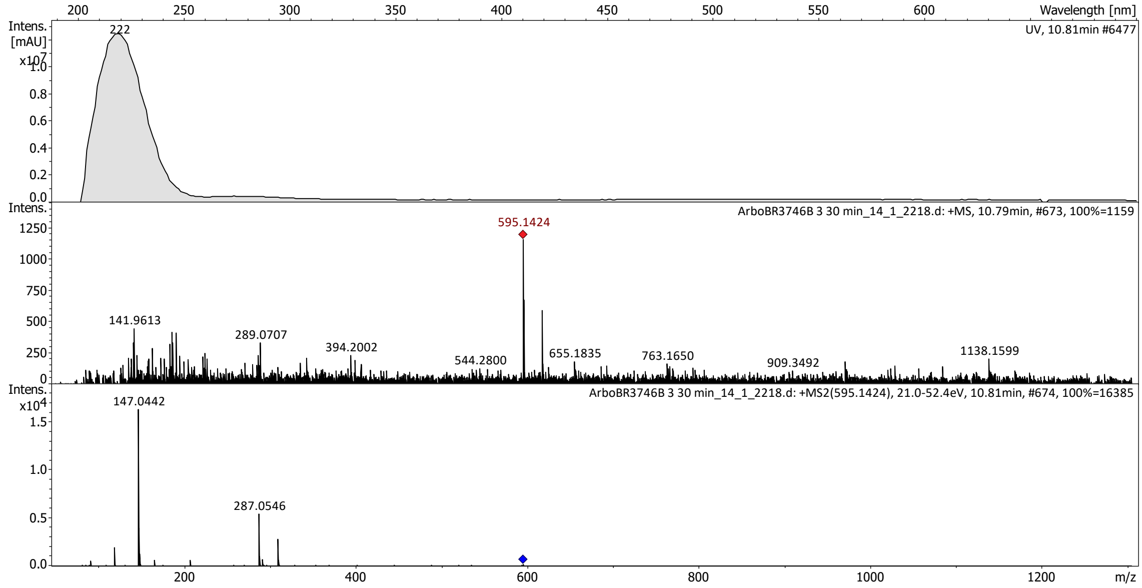

Figure S17. Mass spectra (MS[1] and MS[2]) and UV-VIS spectra of Kaempferol-3-glucoside-6''-*p*-coumaroyl, one of the substances annotated in the hydroethanolic extract of *C. bracteosum*.

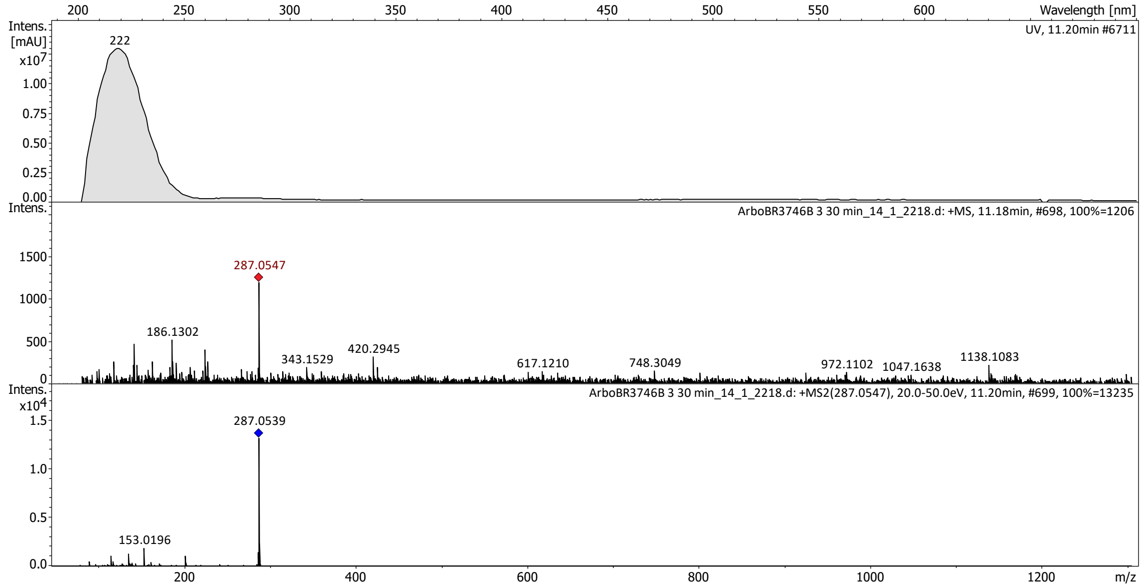

Figure S18. Mass spectra (MS[1] and MS[2]) and UV-VIS spectra of Luteolin, one of the substances annotated in the hydroethanolic extract of *C. bracteosum*.

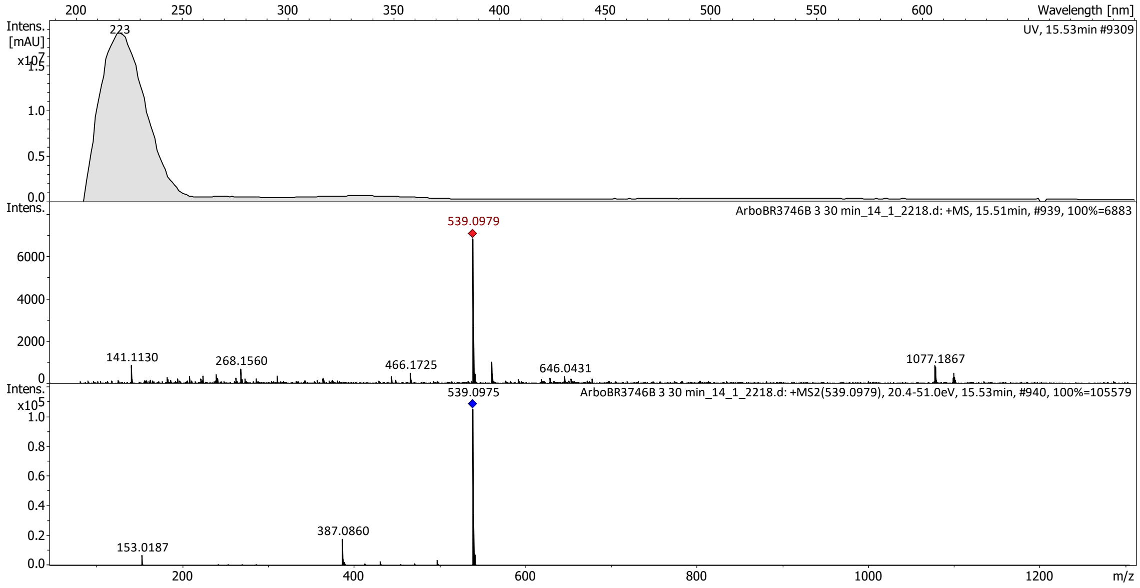

Figure S19. Mass spectra (MS[1] and MS[2]) and UV-VIS spectra of Amentoflavone, one of the substances annotated in the hydroethanolic extract of *C. bracteosum*.

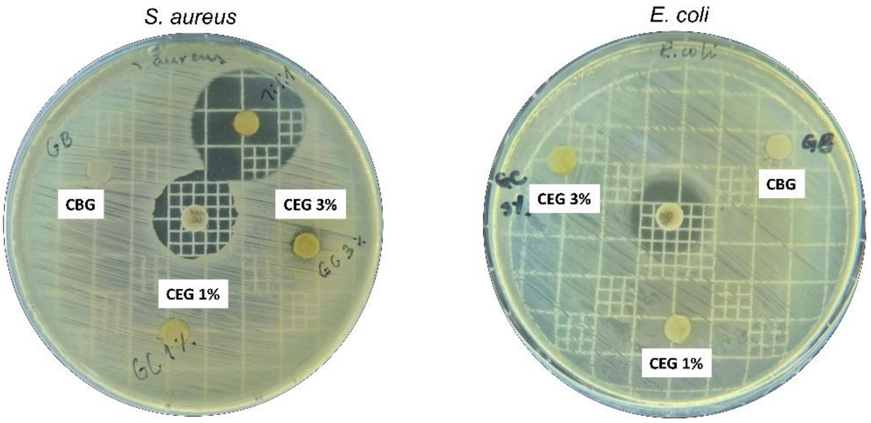

Figure S20. Inhibition halos formed in samples of *C. bracteosum* gels on *S. aureus* and *E. coli*. Sample codes follow the same convention as in the main manuscript: CBG – Carbopol-based gel without extract; CEG1% – Gel containing 1% *C. bracteosum* extract; CEG3% – Gel containing 3% *C. bracteosum* extract. An inhibition zone was observed only for the CEG3% sample (left side of the image).
